# Exploring phenotypic diversity and stability of key traits for apple breeding in northeastern Spanish germplasm

**DOI:** 10.3389/fpls.2025.1623195

**Published:** 2025-09-16

**Authors:** Francisco Javier Bielsa, Pilar Errea, Nerea Iturmendi, Patricia Irisarri, Montserrat Navarro, Lourdes Castel, Jorge Urrestarazu, Luis Gonzaga Santesteban, Carlos Miranda, Ana Pina

**Affiliations:** ^1^ Centro de Investigación y Tecnología Agroalimentaria de Aragón (CITA), Departamento de Ciencia Vegetal, Zaragoza, Spain; ^2^ Instituto Agroalimentario de Aragón-IA2, CITA-Universidad de Zaragoza, Zaragoza, Spain; ^3^ UPNA, Dpto. Agronomía, Biotecnología y Alimentación, Pamplona, Spain; ^4^ UPNA, Instituto de Innovación y Sostenibilidad en la Cadena Agroalimentaria (ISFOOD), Pamplona, Spain; ^5^ UPNA, Instituto de Investigación multidisciplinar en Biología Aplicada (IMAB), Pamplona, Spain

**Keywords:** breeding, collection, germplasm, Malus x domestica Borkh, oxidation, phenotype

## Abstract

*Malus x domestica Borkh* is a key temperate fruit crop globally, but climate change and market demands highlight the need to broaden its narrow genetic base. The collection and conservation of local germplasms support breeding efforts by enabling the development of resilient phenotypes with improved traits. Under this framework, a set of 130 apple accessions (recovered from Northeastern Spain and 14 commercial cultivars were analyzed in terms of 12 phenotypic traits (firmness, SSC, malic acid, enzymatic browning (EB) susceptibility, fructose and glucose content, and phenolic content in peel and pulp). A statistical framework was developed to assess trait stability under shifting climatic conditions and detect significant correlations between climatic variables and phenotypical performance of apple trees. The results revealed a wide phenotypical variation across the studied traits, with the distribution of physicochemical traits strongly influenced in a genotype-dependent manner. A correlation matrix was obtained for studied traits revealing positive correlations between harvest date and SSC, enzymatic browning and firmness. Furthermore, uni- and multifactorial ANOVA revealed that genetic group (GG) is a key factor influencing all measured traits, especially pH, SSC, EB, and browning speed. Several interaction effects were also statistically significant, especially those involving ploidy level, which strongly influenced malic acid content and EB responses. Additionally, significant differences in fructose content were observed depending on peel coloration, and year-to-year phenotypic variation appeared to be predominantly governed by genotypic response to environmental conditions. Moreover, obtention of a mean stability index (D_i_) for each trait allowed the identification of ‘Pinova’,’Fuji’ and ‘Gala’ as the most stable cultivars among 11 commercial cultivars. These findings will support future research on development of functional cultivars and selection of ideal genotypes under shifting conditions.

## 1 Introduction

Apple (*Malus x domestica Borkh*) is a globally cultivated temperate fruit crop with an annual estimated production of 97.33 million tons (FAOSTAT, 2023). Europe contributes significantly to global apple production, producing approximately 17.51 million tons annually, which represents approximately 18% of worldwide apple output. In Spain, apple production has maintained a steady average of 600,000 tons over the last decade, accounting for a substantial share of total national fruit production. Apple versatility, nutritional value, and cultural significance have solidified its place in diets worldwide (Patocka et al., 2020; Sofla et al., 2016). However, despite the great diversity of this species, the global production is dominated by relatively few cultivars, many of which are closely related, leading to genetic erosion (Castel et al., 2020; Cice et al., 2023; Meland et al., 2024; Pina et al., 2014; Sofla et al., 2016). The reason why modern orchards rely on only a few cultivars is due to practical and economic reasons, such as the need for uniformity in fruit quality, high and consistent yields, market preferences, long shelf life, and compatibility with mechanized harvest and post-harvest handling. These factors have contributed to the global dominance of a few elite cultivars, despite their narrow genetic base and vulnerability to biotic and abiotic stresses. This situation has encouraged international coordinated efforts towards the preservation and evaluation of apple genetic resources worldwide (Arnal et al., 2023; Larsen et al., 2024; Mignard et al., 2023; Pina et al., 2014; Tian et al., 2024; Watts et al., 2021). In this sense, an integrative approach was carried out by RosBREED program in USA to study members of the Rosaceae family and the genetic basis of quality and productive traits of stakeholders interest. This project managed to perform a harmonization of Rosaceae members research focuses and implementation of DNA-informed breeding into a more general knowledge that allows a more rapidly evolving improvement of breeding programs (Iezzoni et al., 2020). In Europe, significant progress was also made in the field of apple research within the EU FruitBreedomics project, developing tools/softwares/methodologies and plant materials for the breeders, germplasm curators and scientists as well as deciphering genetic control of main agronomical traits taking into account allelic diversity (Laurens et al., 2018). Other meaningful efforts worth-citing include the improvement of the USDA-ARS National Plant Germplasm System Apple Core Collection in the United States, which comprised an initial set of 3442 diploid accessions and was refined to an optimized core collection of 258 individuals. Additionally, the development of an European reference population (REFPOP) for genomic studies, involving 534 genotypes planted in six different countries, have also provided a valuable phenotypical tool for Genotype x Environment (GxE) studies (Gross et al., 2013; Jung et al., 2020).

Heritage cultivars recovery has been also implemented by germplasm banks worldwide to broaden apple conserved diversity and explore a complete phenotypical landscape in which researchers can find answers as we navigate a rapidly changing climate scenario and new threats arise (Broschewitz et al., 2024; Pereira-Lorenzo et al., 2017; Watts et al., 2021). Local genetic resources offer a broaden phenotypical landscape in which traditional local cultivars and recovered germplasm from abandoned orchards provide new genetic backgrounds that can differ greatly from well-established commercial cultivars. These local genotypes often exhibit improved adaptation to climatic conditions, enhanced conservation traits, extended shelf life and post-harvest performance, as well as health-related qualities such as higher protein and vitamin content, and superior organoleptic properties (Altisent et al., 2014; Millán-Laleona et al., 2023; Moon et al., 2020). Notably, local recovered apple trees are now widely used and have been successfully implemented in apple breeding programs (Cice et al., 2023; Meland et al., 2024; Sofla et al., 2016) providing new insightful research on *Malus domestica* (Ceci et al., 2021; Jalali et al., 2024; Schmitzer et al., 2023a; Zuo et al., 2021).

However, biennial-bearing, climate patterns shifts and other minor statistical variations in horticultural practices, can result in changes of blooming periods, maturation dates and postharvest quality traits (Ameen et al., 2023). This statistically unstable framework can impact the stability of key crop descriptors over time, complicating research efforts and delaying consolidation of breeding program advances. Consequently, the identification and validation of robust stable biomarkers are critical for accurately assessing phenotypic variability within *ex-situ* germplasm collections. Such markers can also aid in the selection of breeding parents that exhibit desirable traits, ensuring the long-term success of breeding programs.

Over the last decades, a heritage recovery approach was implemented in Aragón and Navarra (Spain), retrieving local apple cultivars from mountainous areas, that enriched the apple germplasm collections of the Centro de Investigación y Tecnología Agroalimentaria de Aragón (CITA) and the Universidad Pública de Navarra (UPNA) (Miranda et al., 2018; Pina et al., 2014). In this work, a phenotypic collaborative evaluation was conducted on the CITA and UPNA genebanks, focusing on their respective germplasm collections, which included locally recovered cultivars alongside commercial varieties. These were evaluated as part of an optimized core collection previously assessed using SSR markers (Miranda et al., 2018; Pereira-Lorenzo et al., 2017). To advance in this knowledge, the main objectives of this study were to: i) characterize phenotypical diversity in this core germplasm collection located in Northeastern of Spain, ii) evaluate traits distribution, discriminant power and correlations among them, iii) identify different clusters of cultivars based on phenotypic data and compare them to previous genetic studies, and iv) assess the enzymatic browning indices as potential biomarkers that can account for phenotypic diversity in the collection. A comprehensive statistical framework was developed to establish both the genetic and phenotypic background of the collection, facilitating breeding programs aimed at selecting purpose-driven traits.

## 2 Material and methods

### 2.1 Plant material

Fruit samples were collected from the germplasm collections located in the Universidad Pública de Navarra (UPNA) in Pamplona (42.790380, -1.630360), and from the Centro de Investigación y Tecnología Agroalimentaria de Aragón (CITA) in Zaragoza (41.724351, -0.820441) and Bescós de Garcipollera (42.628189, -0.502759). All the studied locations present a Mediterranean climate, and historical data were recovered from the agroclimatic information system for irrigation (SIAR) (**Supplementary Figure S1**). Mean records for the studied period from April to November during 2020–2023 were for Pamplona 24.41 °C (maximum temperature), 11.33 °C (minimum temperature), 306.35 mm (accumulated precipitation), 16232 MJ/m^2^ (accumulated solar radiation); for Zaragoza 27.94 °C (maximum temperature), 13.00 °C (minimum temperature), 163.16 mm (accumulated precipitation), 18369 MJ/m^2^ (accumulated solar radiation); and for Bescós de Garcipollera 25.28°C (maximum temperature), 10.52°C (minimum temperature), 367.86 mm (accumulated precipitation), 17466 MJ/m^2^ (accumulated solar radiation).

In total, a set of 130 apple accessions and 14 commercial cultivars from 111 genotypes were evaluated (10 commercial apple genotypes and 101 local genotypes cultivated in the three orchards) for 12 basic quality traits: Soluble Solids Content (SSC), Titratable Acidity (TA) and firmness; enzymatic browning index, and harvest date (**Supplementary Table S1**) from 2020 to 2023. Apple orchards were established in a low density open-vase system (4 m × 5 m) and trees were grafted in rootstocks of the commercial M series for apple (MM-106 and MM-111). All trees included in the present study were planted between 5 and 10 years, and yielded at least one full harvest before sampling. Orchards management practices, including fertilization, irrigation and winter pruning, were carried out as in a commercial plantation. The soils at the experimental areas are characterized as heavy and calcareous soils, with a pH from 7.2 to 8.1, organic matter content from 6.9% to 7.9%, active lime content and clay-loam texture. Maturity assessment of apple cultivars was conducted weekly throughout the growing seasons, from early June to late November for late-ripening varieties. The CTIFL scale (2002) was applied to determine the optimal harvest date when cultivars reached an index value of 6–7 (Miranda, 2002). Observations of background colour changes and the presence of dropped apples during field work further guided the selection of the optimal collection date. Not all varieties had fruit available to be collected each year due to biennial bearing habits, spring frosts, or other environmental factors. However, all apple accessions were evaluated at least for two years. The apple accession set has been previously characterized using 13 SSRs (Pereira-Lorenzo et al., 2017) and a core collection was formed that included cider, dessert, processing and heritage apple cultivars (Miranda et al., 2018). The apple collection set, comprising 111 genetic groups was further divided into two subsets (**Supplementary Figure S2**). The first subset included data from years 2020 and 2021, covering 38 genetic groups (36 apple accessions and 13 commercial cultivars), and was used to assess germplasm collection differences and obtain statistical descriptors of studied traits and collection composition. The second subset comprised years 2020 to 2023, covering 24 genetic groups (18 apple accessions and 11 commercial cultivars), and was used to determine correlations with weather variables and assess trait stability, aiming to maximize year range coverage and enhance the robustness of the analysis. Genotypes with fruit data for only one year were excluded from the dataset.

### 2.2 Data collection of firmness measurements, sugar content and acid composition determination

For characterization of total acids and soluble solids content (SSC), juice was extracted by blending 10 apples per variety. The juice phase was decanted, and SSC was determined in triplicate using a refractometer (Pocket Refractometer Pal-1, Atago, Tokyo, Japan). The pH and total acidity expressed as malic acid (g/L) were determined using a titrator (785 DMP Titrino, Metrohm, Herisau, Switzerland) equipped with an automatic sample changer (760-Sample Change, Metrohm, Herisau, Switzerland), employing 0.1N NaOH as the titrant.

Firmness measurements were performed in 10 apples using a penetrometer (Model TR, Turoni, Italy) equipped with an 11 mm plunger tip. Briefly, two peel discs were removed from both sides of each apple around the equatorial plane before firmness was measured.

D-glucose (g/L) and D-fructose (g/L) in apple juice were determined using enzyme assays from commercially available kits (BioSystems, Barcelona, Spain) using a Y15 automatic analyser (BioSystems, Barcelona, Spain). The enzyme assay is based on converting glucose to glucose-6-phosphate (G6P) and fructose to fructose-6-phosphate (F6P) by the hexokinase enzyme. Fructose-6-phosphate is converted to glucose-6-phosphate by the phosphoglucose isomerase enzyme. The glucose-6-phosphate dehydrogenase enzyme oxidizes the total glucose-6-phosphate, generating NADPH that is measured by spectrophotometry. Fructose concentration is determined as the difference in G6P concentration before and after phosphoglucose isomerase treatment.

### 2.3 Total phenolic extraction and quantification

3 g of pulp and 3 g of peel from a pool of 5 apples per variety were frozen in liquid nitrogen. Samples were stored at -80°C until lyophilized using a Lyobeta 4PS freeze dryer (Telstar). The dried samples were weighed and mixed with a methanol solution acidified with 0.05% HCl (v/v) at a ratio of 33.33 (v/w). The samples were sonicated for 15 minutes in an ultrasonic bath, centrifuged at 4000 g for 10 minutes at 6°C, and the supernatant was recovered and stored at -20°C. Phenolic compounds were quantified using the Folin-Ciocalteu reagent following previously described procedure (Millán-Laleona et al., 2023; Teixeira et al., 2023). Samples were incubated for 30 mins and absorbance at 765 nm was measured in a Biochrom Libra S22 spectrophotometer (Biochrom Ltd, Cambridge, UK). A standard curve was prepared with gallic acid (0–2000 mg/mL) and cultivar TPC values were expressed in mg of gallic acid equivalent per 100 g of sample (mg GAE/100 g fresh weight sample).

### 2.4 Enzymatic browning indexes determination

The enzymatic browning capacity of apple pulp halves from each germplasm collection was evaluated during the 2020 and 2021 growing season. Digital image analysis was conducted at oxidation time points of 0, 30, and 60 minutes. Enzymatic browning was calculated as the difference in browning index (ΔBI) from the time of cutting as ΔBI = BI_0_ - BI_t_ (ΔBI 0–30 and ΔBI 0–60) along with oxidation speed index SEB_ΔBI as oxidative characteristics at the Universidad Pública de Navarra, enabling classification of accessions into clusters with varying oxidation susceptibility levels (Miranda et al., 2023).

### 2.5 Statistical analyses

All analysis used Python version 3.11 and Spyder version 5.4.3, leveraging Python packages such as NumPy, pandas, matplotlib, seaborn and scikit-learn. Several statistical tests were carried out to determine genotype behaviour across multiple factors (year, geographic origin, colour) and to establish significant differences and ranges among the studied cultivars and traits. Trait normality was assessed with the Shapiro-Wilk test and variance homogeneity was verified using Levene’s test. For each quantitative variable, differences among categorical groups (year, origin and colour) were tested using one-way Analysis of Variance (ANOVA). *Post-hoc* comparisons (Tukey’s for homoscedastic data or Dunn’s tests for heteroscedastic data) were employed to determine which specific groups differed when the ANOVA indicated a significant effect (p<0.05). When normality assumptions were not met, the Kruskal-Wallis test was applied, supplemented by Dunn’s *post-hoc* tests for pairwise comparisons.

Furthermore, the AMMI (Additive Main Effects and Multiplicative Interaction) model was employed to study genotype x year (GxY) interaction following previous procedures (Liu et al., 2017; Ye et al., 2021). The AMMI model was defined as follows:


$$y_{i,j}=\;\mu +\;\alpha_{i}+\;\beta_{j}+\;\sum_{k=1}^{K}\lambda_{k}\gamma_{i,k}\delta_{j,k}+\epsilon_{i,j}$$

Where $y_{i,j}$ was the observed performance of genotype i in year j, μ is the overall mean, $\alpha_{i}$ ​ and $\beta_{j}$ are the main effects of genotype i and year j, respectively, and the term $\lambda_{k}\gamma_{i,k}\delta_{j,k}$ represents the multiplicative interaction captured by the first K singular values and vectors. One of the major benefits of AMMI is that it provides a quantitative way to assess genotype stability. Specifically, each genotype has loadings $\gamma_{i,k}$ ​ on the interaction principal components (IPCAs). If a genotype has large loadings on the first few IPCAs, it indicates that its performance fluctuates more across environments. Conversely, small loadings imply more stable performance. Stability index (Di) was calculated as follows for the three first IPCAs:


$$D_{i}=\;\sqrt{\sum_{r=1}^{C}{\left(\gamma_{i,r}\sqrt{\sigma_{r}}\right)}^{2}}$$

$\gamma_{i,r}$ is the genotype i loading on the r-th IPCA, $\sigma_{r}$ is the variance (singular value squared) of that component and C is the number of components retained.

To evaluate each parameter’s performance, we calculated the Coefficient of Variation (CV) to quantify the relative variability of each trait with respect to its mean value across genotypes,


$$CV=\;\frac{s}{\bar{x}}$$

where s the standard deviation of the trait and $\bar{x}$ is its mean across genotypes, thus indicating relative variability.

Detection of genetic diversity of studied apple germplasm was done using Shannon-Wiener genetic diversity index (H’) evaluating biochemical parameters as previously done (Tian et al., 2024). Briefly, H’ was defined as:


$$H’=\;\sum_{i=1}^{m}p_{i}ln(p_{i})$$

where $p_{i}$ is the proportion of observations in the i-th category, capturing how trait values are distributed among genotypes. Likewise, discriminant ability for each trait was assessed using two complementary metrics: Discriminant Power (DP) and Discriminant Ratio (DR) (Levy et al., 1999) following:


$$DP=\;\frac{S_{b}}{\bar{S_{total}}}$$

$$DR=\;\frac{\sqrt{s_{b}^{2}-\frac{S_{w}^{2}}{k}}}{S_{w}}$$

where $S_{b}$ ​ is the standard deviation of the genotype means, $S_{total}$ is the overall standard deviation, $S_{w}$ is the average within-genotype standard deviation, and k is the average number of replicate measurements per genotype. Evaluation of genotype-trait stability, discriminant power, discriminant ratio, correlation coefficients and colour analysis were performed on a reduced dataset of 30 unique accessions evaluated during 4 consecutive years (2020-2023).

Moreover, correlations between studied traits was evaluated using Pearson’s correlation coefficient using the corr() test function in python. Principal Component Analysis (PCA) was also performed for data reduction and assessment of trait loadings in first components to determine specific weight of each trait in data segregation. In parallel, K-means clustering was employed, with the number of clusters chosen by referencing both the elbow method and the silhouette analysis. By combining these two approaches, we achieved a balanced assessment that accounts for both intra-cluster homogeneity and inter-cluster separation, yielding a robust clustering solution. Finally, climatic data was obtained from national Spanish database of the Agroclimatic Information System for Irrigation (SiAR), using meteorological records from the available station closest to the orchards. A correlation analysis between biochemical traits and climate variables was then performed using Pearson’s correlation coefficient using the corr() test function in python.

## 3 Results

### 3.1 Physicochemical and biochemical trait variation on the apple collections (CITA and UPNA)

**Figure 1:**
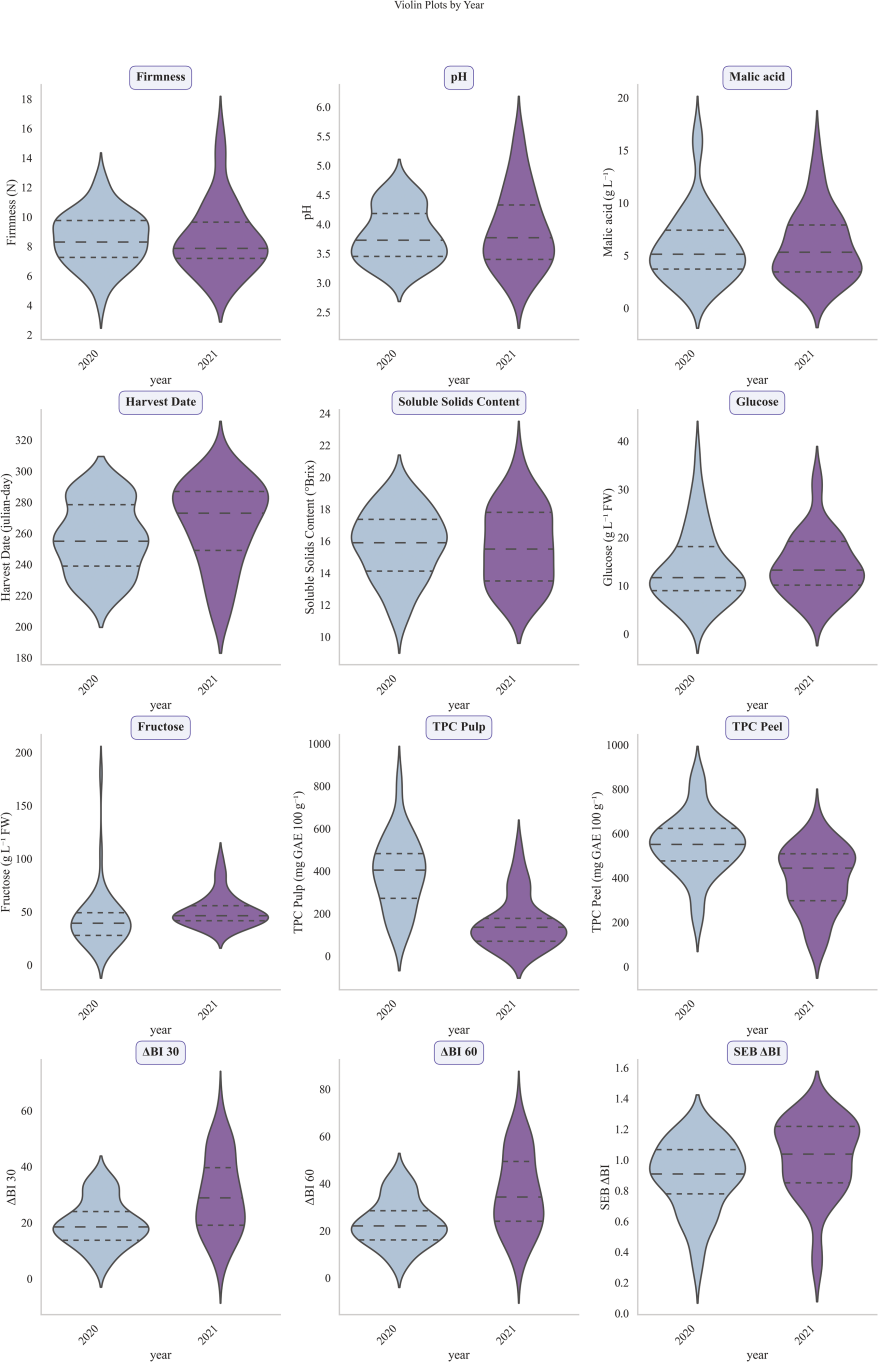
Violin plots of 12 studied traits from two different germplasm collections (CITA and UPNA). From top to bottom and left to right studied traits were firmness (N), pH, malic acid (g/L), harvest date (Julian days), soluble solids content (°Brix), glucose (g/L), fructose (g/L), total phenolic content (TPC) in pulp (mg GAE/100 g FW), TPC in peel (mg GAE/100 g FW), differences in browning index at 30 and 60 min (ΔBI 30, ΔBI 60) and oxidation speed index (SEB ΔBI).

The evaluation of quality traits showed that both collections exhibited similar ranges, with only small, non-significant mean differences from 2020 to 2021 (**Supplementary Table S1**, **Figure 1**). These differences can be attributed to variations in the representation of cultivar genetic groups, given that the CITA collection included a greater number of samples than the UPNA collection. Across both collections, pH ranged from 2.86 (‘Yosa de Sobremonte_01’) to 5.55 (‘Golden Delicious’), while malic acid levels varied from 1.22 g/L (‘Troncedo_01’) to 17.01 g/L (‘Cenarbe_07’). There was an approximate four-fold difference in firmness among accessions at harvest: the softest accession was ‘Santa Eulalia_01’ with a measurement of 4.14 kg/cm^2^, whereas the firmest accession was ‘Javierre del Obispo_02’ at 15.89 kg/cm2. Mean firmness among the collection was 8.51 kg/cm^2^, slightly higher than recommended consumer preferred firmness of 6–7 kg/cm^2^, highlighting a slight influence of local firmer cultivars on the global pool. Soluble solids content (SSC), which approximates sugar concentration, reached a maximum of 21.20°Brix (‘Pomera de Pomes agrias’) and a minimum of 10.93°Brix (‘Manzana Tomate’). Individual analyses of glucose and fructose revealed glucose levels ranging from 3.05 g/L (‘Pink Lady’) to 37.10 g/L (‘Barcabo_01’), and fructose levels ranging from 13.15 g/L (‘Torres Albarra_02’) to 180.35 g/L (‘Reineta Gris’).

Finally, the quantification of total phenolic content (TPC) revealed a broad range in both pulp and peel, likely due to the overrepresentation of local cultivars in the collection. Pulp TPC ranged from 12.7 (‘De Mine’) to 826.9 mg/100 gFW (‘Manzana amarrilla de octubre’), whereas peel TPC ranged from 85.8 (‘Royal Gala’) to 863.95 (‘Goikoetxe’) (**Figure 1**).

**Figure 2:**
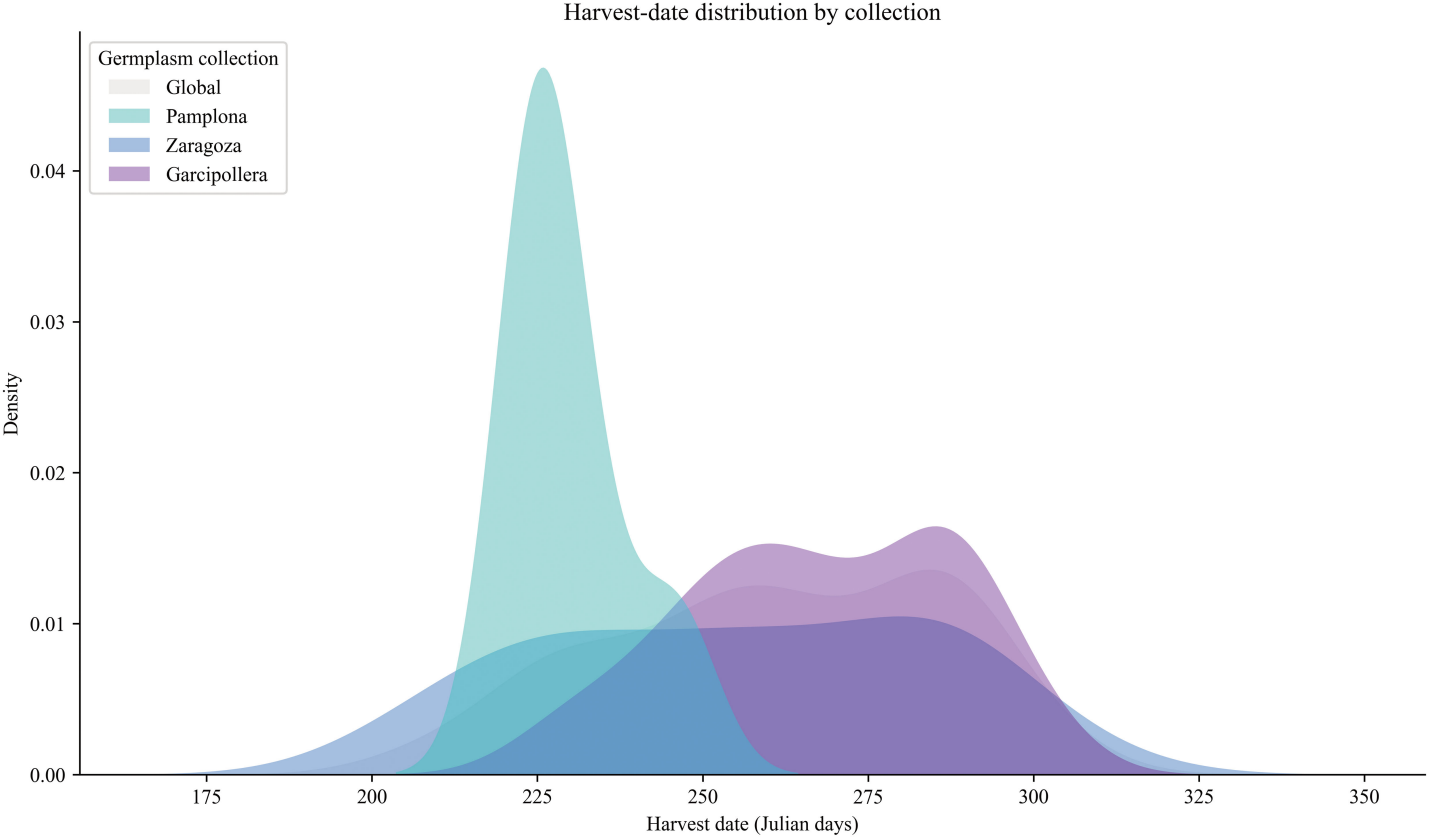
Harvest-date distribution by collection. Distribution of harvest date expressed as Julian days among the different germplasm collections during harvest seasons 2020 and 2021. A global trend is shown in grey, UPNA collection corresponds to Pamplona (in cyan), CITA collections corresponds to Zaragoza (in dark blue) and Bescos de la Garcipollera (in purple).

**Figure 3:**
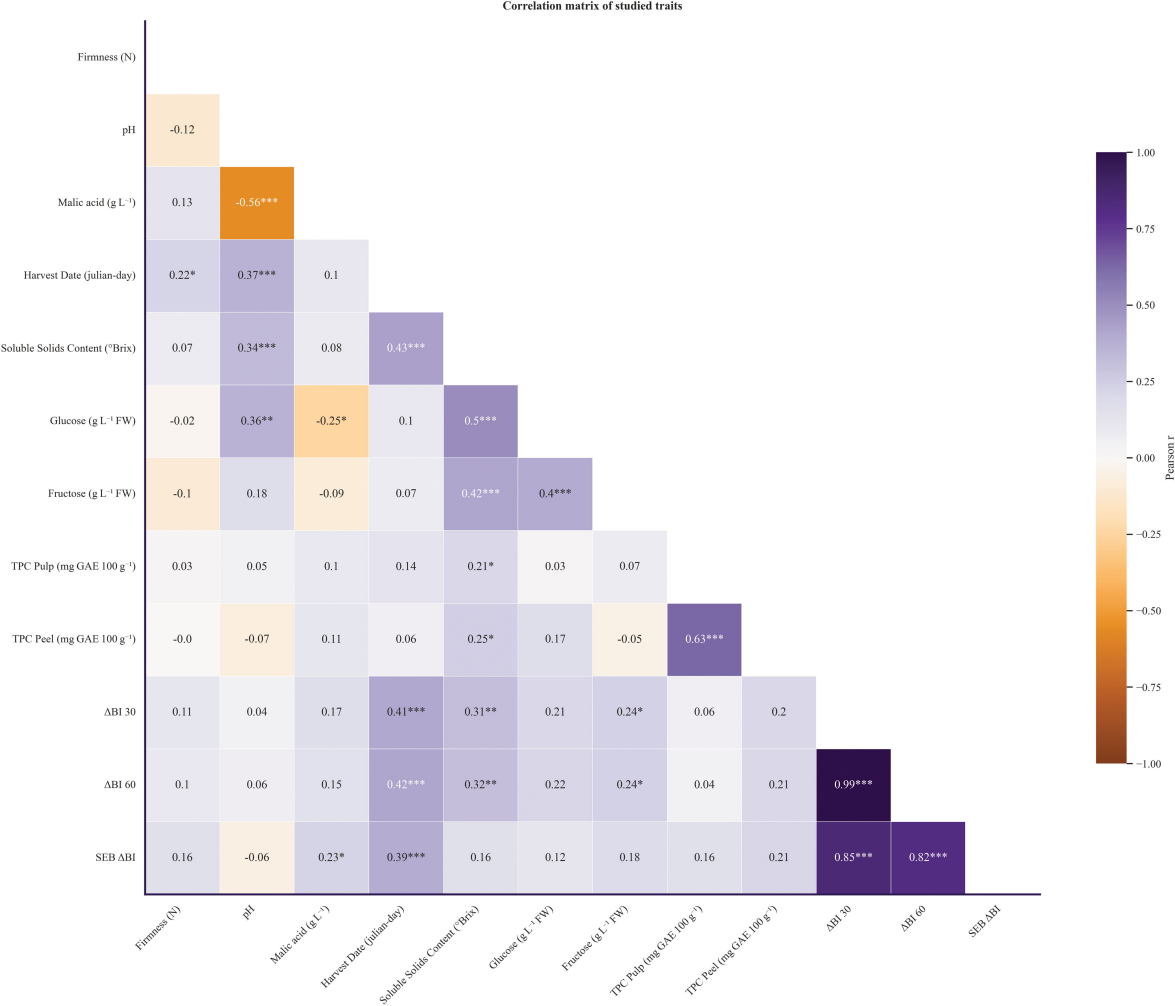
Correlation Matrix of studied traits using Pearson correlation index. Positive values are shown in violet whereas negative values are marked with orange. Significant p-values are marked with *, ** and *** for levels of significance 0.05, 0.005 and 0,001, respectively.

To further characterize our apple collections (CITA and UPNA), harvest dates of our cultivars were recorded in Julian days. Furthermore, the relationship between quality traits and maturation dates was assessed. A simple distribution representation revealed that our collection is somewhat biased toward late-ripening apple trees (**Figure 2**), which also exhibit higher values of pH, SSC, enzymatic browning index (ΔBI) and firmness that are correlated with harvest time (**Figure 3**).

### 3.2 Correlation analysis of phenotypic traits and principal component analysis

A correlation matrix was obtained calculating pearson’s correlation coefficient between 12 studied traits from 2020–2021 dataset. Strong correlations (> 0.80, p < 0.001) were observed between oxidation indexes ΔBI at 30 and 60 minutes and oxidation speed index SEB_ΔBI (**Figure 3**), as it was expected. However, when comparing their interactions with biochemical traits, oxidation indexes performed unexpectedly, showing no correlation between TPC in pulp and ΔBI values at 30 and 60 minutes of oxidation. Instead, ΔBI correlated positively with SSC (r = 0.3, p < 0.005). Surprisingly, speed oxidation rate SEB_ΔBI also correlated positively with malic acid content (r = 0.23, p < 0.05). In addition, both SEB_ΔBI and ΔBI values at 30 and 60 minutes correlated positively (r = 0.39, r = 0.41, r = 0.3, respectively, p < 0.001) with harvest date, pointing out that late-varieties tended to develop enzymatic browning faster and more notably. Harvest date also correlated positively with SSC (r = 0.43, p < 0.001), which was also related to ΔBI, indicating a strong relationship between late-harvest varieties with higher SSC and enzymatic browning susceptibility. Moreover, harvest date was also correlated positively with firmness (r = 0.22, p <0.05) which indicated that firmness was also higher for late ripening varieties (**Figure 3**).

Likewise, significant strong positive correlations were found between SSC and glucose and fructose (r = 0.50 and r = 0.42 respectively, p < 0.001), which also showed moderate positive correlations with pH (r = 0.34, p < 0.005), and TPC in peel (r = 0.25, p < 0.05) and pulp (r = 0.21, p < 0,05). Malic acid content, as it was expected, correlated negatively with pH (r = -0.56, p < 0.001) and glucose (r = -0.26, p < 0.05). Finally, glucose and fructose correlated positively with each other (r = 0.4, p < 0.001) and TPC in pulp and peel also showed a strong positive correlation (r = 0.63, p < 0.001) (**Figure 3**).

**Figure 4:**
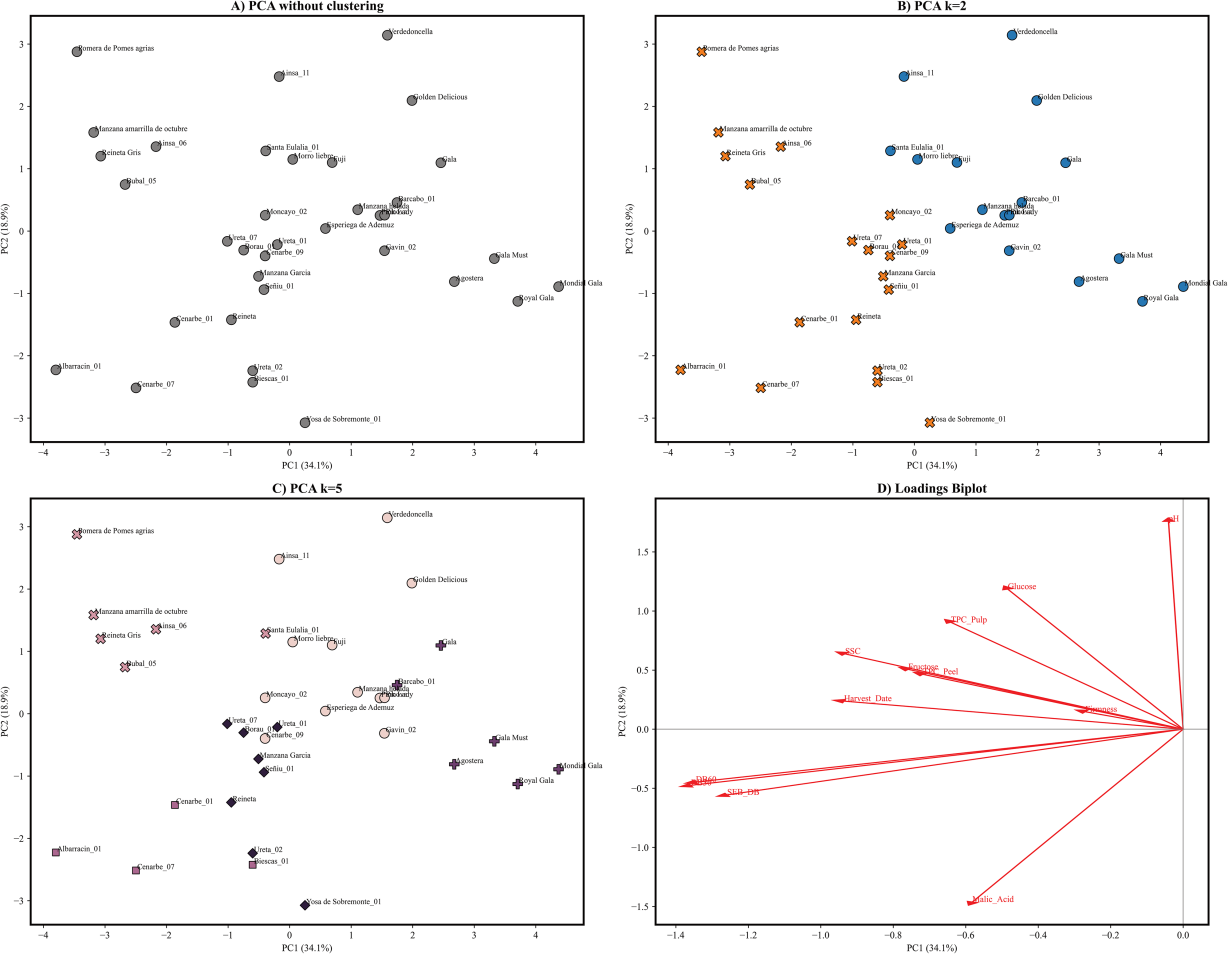
**(A)** PCA results based on the measurement of 12 physicochemical and biochemical traits in 36 apple genotypes collected during 2020-2021. PC1 explained 34.1% of the variance whereas PC2 explained 18.9% of the variance. **(B)** PCA with coloured membership of obtained groups after clustering using k means for k=2, groups 1 and 2 are marked orange and blue. **(C)** PCA with coloured membership of obtained groups after clustering using kmeans for k=5, groups 1,2,3,4 and 5 are coloured in lightpink, pink, light purple, purple and black. **(D)** Biplot of PCA loadings in first and second principal components, a longer arrow represents a higher loading (interaction) in principal component towards the arrow is pointing. Lowest interaction is shown by firmness, whereas high interactions are shown by oxidation indexes ΔBI.

**Figure 5:**
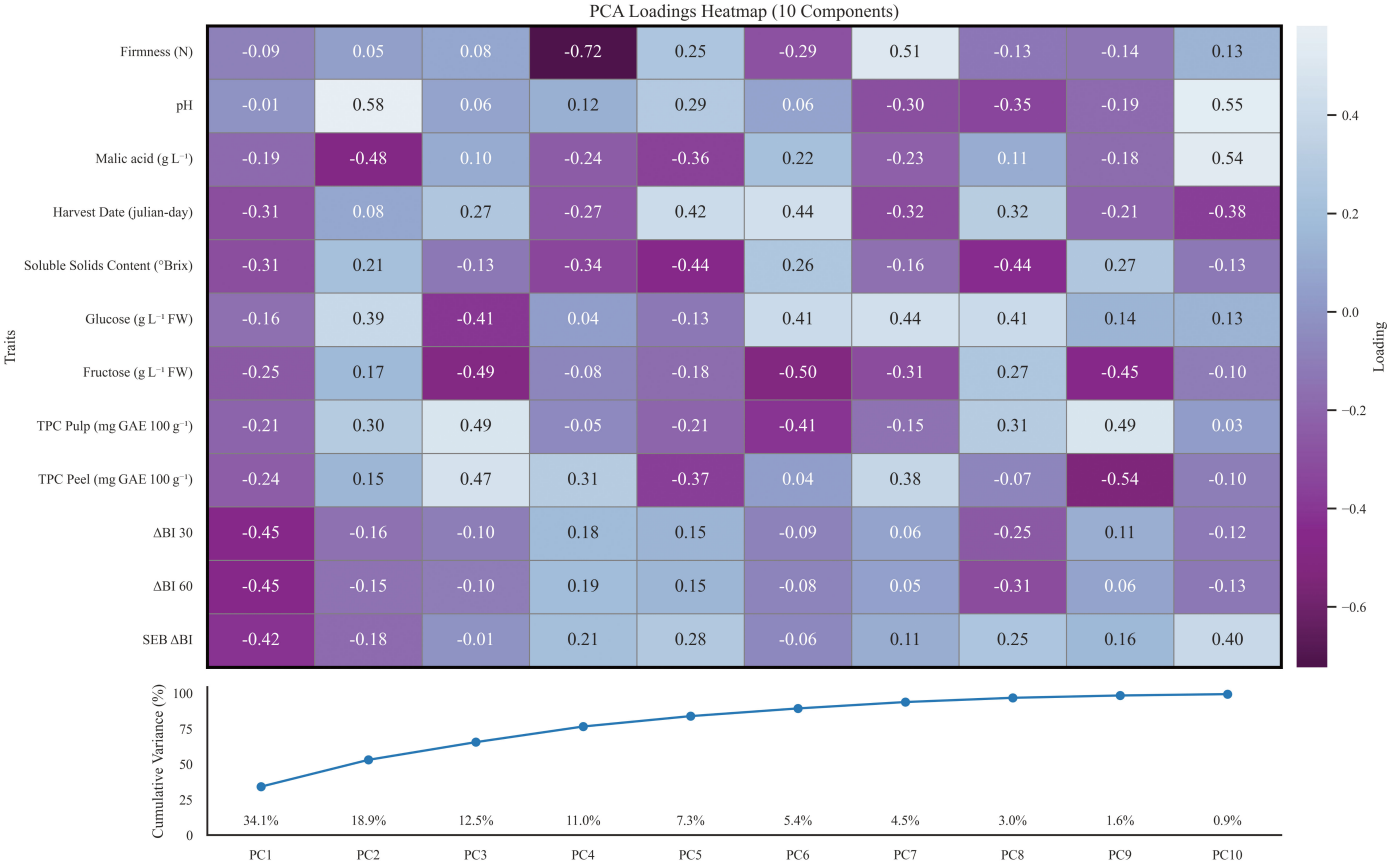
Top: PCA loadings heatmap of all studied traits for 10 first principal components. Positive loadings are coloured blue, whereas negative loadings are coloured purple. Principal components are shown in order from first column to tenth. Bottom: Accumulated explained variance by individual principal components of the PCA. Each component’s explained variance is plotted in the x axis whereas the dot line shows the accumulated variance for the sum of previous principal components.

Furthermore, a principal component analysis (PCA) was conducted to evaluate the distances between all the studied apple genotypes based on the measured fruit attributes, aiming to understand how these traits contribute to the differentiation of the accessions (**Figure 4**). In order to perform the principal component analysis, data was previously curated to avoid missing data and assure cultivar representation over all selected years, making a total of 36 genotypes. Principal component analysis of 2020–2021 data achieved > 80% explained variance with 5 or more components. PC1 and PC2 accounted for 34.1% and 18.9% of the total variability, respectively. PC1 loadings were mainly given by oxidation trait indexes that contributed with a negative load between -0.42 (SEB_ΔBI) to -0.45 (ΔBI) (**Figure 5**), pointing out that a wide range of phenotypical diversity can be captured by oxidation traits (**Figure 4**). PC1 lesser loadings were given by harvest date (-0.31), malic acid (-0.31) and SSC (-0.31). Contrarily, PC2 loading were dominated by malic acid (-0.48) in the negative loadings and pH (0.58), glucose (0.39) and TPC in pulp (0.30) in the positive loadings. PC3 contributed with a moderate 12.50% of the explained variance. The variables contributing most strongly to this component include TPC in pulp (0.49) and peel (0.0.47), fructose (-0.49), glucose (-0.41) and harvest date (0.27). PC4 loadings ranged from -0.72 (firmness) to 0.31 (TPC in peel) and explained an additional 11% of the variance. The remaining components each contributed less than 8% to the total variance and reflected residual loadings of studied traits. Overall, the analysis reveals that oxidation trait ΔBI at 30 and 60 minutes, and its respective oxidation speed rate SEB_ΔBI can account for a great amount of variance in our collection, followed by acid and sugars and in a lesser count by TPC and firmness.

Moreover, a hierarchical clustering was made based on physicochemical traits allowing identification of cultivar groups for k = 2 and k = 5 (**Figure 4**). Using k-means resulted in the selection of k = 2 as an optimal k. However, a more thorough analysis using silhouette scores and elbow method allowed the visualization of probable subdivisions. A total of 25 accessions and 11 commercial cultivars were plotted in a PCA representation, also showing cluster membership for k = 2 and k = 5. Hierarchical clustering using k = 2 revealed a cultivar division into a first group of 17 cultivars mainly populated by well stablished commercial cultivars such as ‘Royal Gala’, ‘Golden Delicious’ or ‘Pink Lady’ and 7 locally recovered cultivars. Contrarily, the second group contained 19 accessions, 17 local cultivars and only two triploid commercial cultivars ‘Reineta’ and ‘Reineta gris’. Cluster 1 was positioned on the right side of the PCA1 (**Figure 5**), associated with low oxidation level and low TPC in pulp and peel whereas Cluster 2 was located on the left-side, grouping cultivars with higher oxidation level, later harvest dates and increased SSC and acid content. Subsequent subdivision using k = 5, separated Cluster 1 into two different subgroups and Cluster 2 into three. Within Cluster 1, one subgroup consisted mainly of ‘Gala’ type cultivars with low oxidation and equilibrated sugar and acid content, while the second subgroup showed greater dispersion among the remaining accessions. Moreover, subdivision of Cluster 2 was mainly driven by acid content and TPC in pulp and peel, generating a subgroup on the lower portion of the PC2 that comprised 7 local cultivars along with ‘Reineta’. Two additional subgroups emerged on the upper side of the PCA: one composed of equilibrated cultivars and another consisting of ‘high value’ cultivars with extreme values of sugars and phenolic content in both pulp and peel. The clustering analysis with k = 5 revealed a more heterogeneous subgroup composition compared to k = 2, accounting for phenotypical variability comprised in local cultivars and enabling the identification of well differentiated groups with similar trait distribution. In summary, oxidation indexes along with sugar and acid content have a significant impact on accession grouping. These variables, together with total phenolic content (TPC), provide sufficient discriminatory power to clearly distinguish between reference cultivars and local recovered cultivars.

### 3.3 Trait discriminant power and factor influence on distribution determination using ANOVA

In order to elucidate the most discriminant trait, determination of discriminant ratio (DR) (Levy et al., 1999) and discriminant power (DP) was analyzed. While CV and H′ reflect the consistency and diversity of trait values across genotypes, DP and DR indicate how effectively each trait discriminates the genotypes under study. The results revealed that malic acid was the most discriminant trait among 12 studied variables showing highest DP (0.97) and DR (3.42) values, giving the best discriminant scores for studied parameters (**Table 1**). Contrarily, fructose content, TPC in pulp and TPC in peel showed the lowest discriminant power values (0.70, 0.77 and 0.80 respectively), obtaining also lowest DR values (0.59, 0.51 and 0.82 respectively). Oxidation traits showed discriminant ratios ranging from 1.19 (ΔBI30) to 1.39 (SEB_ΔBI), positioning on the lower percentile of studied traits. Firmness, harvest date, SSC and pH obtained moderate discriminant values ranging from 1.54 to 1.72 in DR and from 0.90 to 0.91 in DP. Overall, the collection showed diversity values greater than 1.69 for all studied parameters. Greatest diversity values were obtained for SSC (H’ = 2.08), TPC in peel (H’ = 2.03) and firmness (H’ = 2.00).

**Table 1:** Statistical main descriptors of studied traits among the germplasm collections.

Trait	Mean	Min	Max	SD	CV	H’	Di	DP	DR
*Firmness*	8,52	4,15	15,90	2,11	0,25	2,01	0,00	0,90	1,72
*pH*	3,85	2,86	5,55	0,57	0,15	1,97	0,00	0,88	1,61
*Malic Acid (g/L)*	5,89	1,22	17,01	3,38	0,57	1,87	0,00	0,98	3,43
*Harvest Date*	261,21	208,00	307,00	25,02	0,10	2,00	0,02	0,92	1,64
*SSC (°Brix)*	15,74	10,93	21,20	2,29	0,15	2,08	0,00	0,91	1,54
*Glucose (g/L)*	13,94	3,05	37,10	6,99	0,50	1,89	0,01	0,81	1,00
*Fructose (g/L)*	46,27	13,15	180,35	23,19	0,50	1,69	0,03	0,71	0,60
*TPC Pulp (mg GAE/100 g FW)*	277,40	12,70	826,90	189,23	0,68	1,90	1,00	0,78	0,52
*TPC Peel (mg GAE/100 g FW)*	474,84	85,80	863,95	161,94	0,34	2,03	1,00	0,80	0,82
*ΔBI 30*	24,97	3,99	61,32	12,49	0,50	1,97	0,02	0,83	1,20
*ΔBI 60*	29,94	4,57	72,35	15,03	0,50	1,96	0,03	0,83	1,17
*SEB ΔBI*	0,95	0,28	1,35	0,25	0,26	1,96	0,00	0,87	1,40

Trait discriminant ability, diversity and stability over 2020–2023 for 18 apple accessions and 11 commercial cultivars. Mean, mean value; Min, minimum recorded value; Max, Maximum value; SD, Standard deviation; CV, Coefficient of variation; H’, Shannon’s diversity index; DP, Discriminant power; DR, Discriminant Ratio.

**Figure 6:**
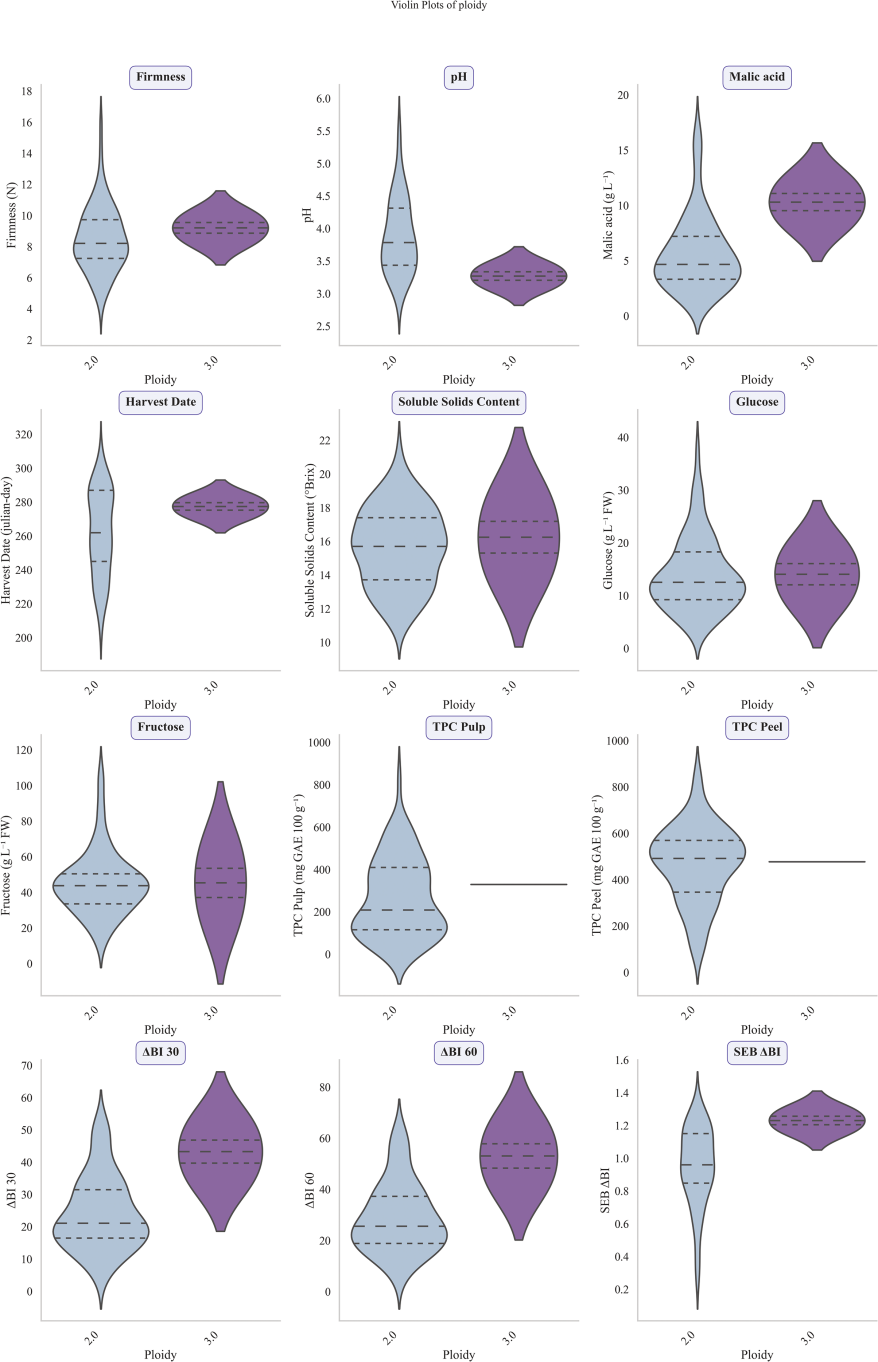
Violin plots of 12 phenotypic traits for 36 apple genetic groups across ploidy levels for the 2020–2021 growing seasons. From top to bottom and left to right studied traits were firmness (N), pH, malic acid (g/L), harvest date (Julian days), soluble solids content (°Brix), glucose (g/L), fructose (g/L), total phenolic content (TPC) in pulp (mg GAE/100 g FW), TPC in peel (mg GAE/100 g FW), ΔBI 30, ΔBI 60 and SEB ΔBI. Values from diploid accessions are shown in blue whereas values from triploid accessions are in purple.

Determination of factor influence on trait distribution was performed by One-way ANOVA. Studied categorical factors were ‘year’, ‘geographic origin’, ‘colour’ (determined in previous works according to Royo et al. (2017)) and ‘ploidy’ (Pina et al., 2014; Urrestarazu et al., 2012; Pereira-Lorenzo et al., 2017). This procedure was applied to a complete set of 36 apple genotypes (UPNA and CITA) during years 2020–2021 and a reduced set comprising 24 genetic groups from CITA collected over an extended period of 4 years (2020-2023) obtaining similar results. Factor ‘year’ showed significant values (p< 0.05) for TPC in pulp and peel (F = 51.52 and F = 27.21, respectively) (**Table 2**). Although the impact of ‘colour’ in studied traits was lower, it affected a greater numerous of variables, showing a significant influence on SSC, harvest date, fructose, ΔBI 30, ΔBI 60 and SEB_ΔBI. F-values ranged from F=2.47 for SSC to 6.08 for fructose (**Table 2**). The geographical origin of the accessions also had an impact on ΔBI 30 and ΔBI 60, highlighting the differences in enzymatic browning between commercial cultivars harvested from valley areas and local cultivars from mountain zones. Finally, ploidy effect assessment revealed significant values for malic acid content and oxidation indexes ΔBI 30, ΔBI 60 and SEB_ΔBI. Triploid accessions (2.60% of the collection) had higher values of malic acid (F= 3.58) and were more susceptible to enzymatic browning (**Figure 6**).

**Table 2:** Unifactorial ANOVA results for the 2020–2021 dataset.

Variable	Factor	F	p-value
*TPC Pulp*	year	51,52	0,0000
*TPC Peel*	year	27,22	0,0000
*ΔBI 30*	year	17,72	0,0001
*ΔBI 60*	year	19,65	0,0000
*SEB ΔBI*	year	5,84	0,0176
*Harvest Date*	colour	2,55	0,0456
*Fructose*	colour	6,09	0,0003
*ΔBI 30*	colour	3,41	0,0126
*ΔBI 60*	colour	3,23	0,0164
*SEB ΔBI*	colour	2,99	0,0236
*ΔBI 30*	origin	4,52	0,0053
*ΔBI 60*	origin	5,01	0,0029
*ΔBI 30*	ploidy	5,47	0,0218
*ΔBI 60*	ploidy	6,04	0,0161
*SEB ΔBI*	ploidy	2,78	0,0994

F-values and p-values are provided for both cases. From left to right: Variable which is affected by the studied factor, evaluated factor, f-value and p value. Each row shows the quantitative effect of a certain factor (year, colour, origin and ploidy) to a specific trait and its level of significance.

Univariate analysis was combined with multivariate analysis to try to capture the more complex relationships that occur in a real field situation in which the effects of factors may be modulated by other factors. This multifactorial analysis included previous factors (year, origin, colour and ploidy) and ‘genetic group’ to evaluate genotype-dependent effect of trait response for each variable. A significant main effect of factor ‘genetic group’ was detected and showed highly significant effects on all physicochemical traits with an F-value ranging from 9.33 in TPC in pulp to 787.27 in SSC (**Table 3**). The interaction effects among the factors ‘genetic group’, ‘colour’, ‘origin’ and ‘ploidy’ were also assessed in this analysis. Different combinations of these factors revealed significant interaction effects, indicating that several traits are modulated by the presence of a second factor (**Table 3**). For instance, SSC and TPC in pulp showed a significant effect of origin and colour modulated by ‘genetic group’, highlighting that differences in these traits can be attributed not only to the geographical origin of the cultivars or apple surface colour, but also to the specific environmental responses associated with each genotype. A similar combination of effects was also observed in malic acid content and oxidation indexes, where significant effects were driven by the interactions ‘colour:ploidy’ and genetic ‘group:ploidy’. These results indicate that differences between varieties with differing colour or genetic background were more pronounced in triploid cultivars, suggesting that ploidy level intensifies the influence of other factors on these traits.

**Table 3:** Multifactorial ANOVA results for two consecutive years 2020-2021. .

Factor	sum_sq	df	F	PR(>F)	Variable
*C(gg)*	13929,18	37,00	120,72	0,0000	Firmness
*C(gg)*	4401,07	37,00	488,10	0,0000	pH
*C(gg)*	7282,07	37,00	43,99	0,0000	Malic Acid
*C(colour):C(gg)*	1713,02	148,00	2,59	0,0432	Malic Acid
*C(colour):C(ploidy)*	142,55	4,00	7,97	0,0154	Malic Acid
*C(origin):C(gg)*	1962,39	111,00	3,95	0,0075	Malic Acid
*C(origin):C(ploidy)*	106,91	3,00	7,97	0,0154	Malic Acid
*C(gg):C(ploidy)*	1318,60	37,00	7,97	0,0154	Malic Acid
*C(gg)*	86318,88	37,00	787,28	0,0000	SSC
*C(colour):C(origin)*	120,97	12,00	3,40	0,0444	SSC
*C(colour):C(gg)*	871,20	148,00	1,99	0,1058	SSC
*C(origin):C(gg)*	700,82	111,00	2,13	0,0829	SSC
*C(gg)*	33712,40	37,00	32,51	0,0023	Glucose
*C(gg)*	2449831,95	37,00	9,34	0,0137	TPC Pulp
*C(colour):C(gg)*	4393145,84	148,00	4,19	0,0150	TPC Pulp
*C(origin):C(gg)*	2901096,54	111,00	3,69	0,0223	TPC Pulp
*C(gg)*	98843542,15	37,00	161,88	0,0000	TPC Peel
*C(gg)*	202514,57	37,00	111,42	0,0000	ΔBI 30
*C(colour):C(ploidy)*	1458,99	4,00	7,43	0,0234	ΔBI 30
*C(origin):C(ploidy)*	1094,24	3,00	7,43	0,0234	ΔBI 30
*C(gg):C(ploidy)*	13495,65	37,00	7,43	0,0234	ΔBI 30
*C(gg)*	345204,65	37,00	116,31	0,0000	ΔBI 60
*C(colour):C(ploidy)*	2281,79	4,00	7,11	0,0258	ΔBI 60
*C(origin):C(ploidy)*	1711,34	3,00	7,11	0,0258	ΔBI 60
*C(gg):C(ploidy)*	21106,55	37,00	7,11	0,0258	ΔBI 60
*C(gg)*	295,61	37,00	414,59	0,0000	SEB ΔBI
*C(year):C(colour)*	0,46	4,00	5,94	0,0162	SEB ΔBI
*C(colour):C(gg)*	9,46	148,00	3,32	0,0320	SEB ΔBI
*C(colour):C(ploidy)*	0,58	4,00	7,52	0,0228	SEB ΔBI
*C(origin):C(gg)*	7,45	111,00	3,48	0,0269	SEB ΔBI
*C(origin):C(ploidy)*	0,43	3,00	7,52	0,0228	SEB ΔBI
*C(gg):C(ploidy)*	5,36	37,00	7,52	0,0228	SEB ΔBI

F-values and p-values are provided. Factor: factor interaction combination, sum_sq: sum of squares, df: degrees of freedom, F-value, PR(>F): p-value, variable. Each row shows the quantitative effect of a certain factor (year, colour, origin and ploidy) or combination of factors (f.e. colour:gg) to a specific trait and its level of significance. Factors; gg, genetic group (based on previously obtained SSR data), colour (red, green, yellow, bicoloured, brown), origin (Pamplona, Zaragoza, Garcipollera), ploidy (2-3).

### 3.4 Additive main effect multiplicative interaction analysis and climate variability correlation analysis

**Figure 7:**
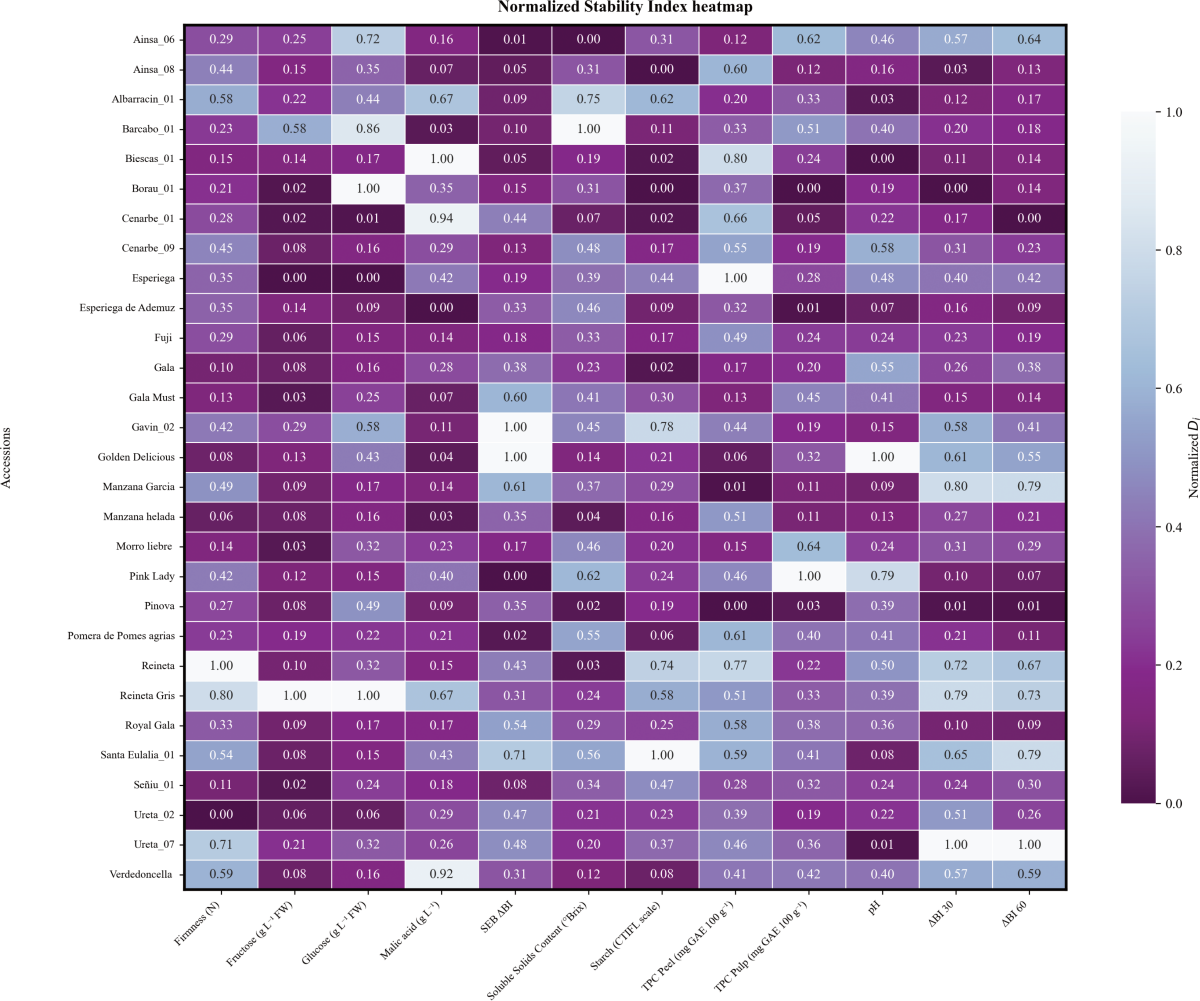
Normalized heatmap of the AMMI Stability Index (Di) for all studied accessions during 4 consecutive years, from 2020 to 2023. Studied traits are shown in the x axis; accessions are plotted as rows in the y axis. Darker colours represent lower stability index values (higher stability for a certain trait). Lighter colours represent higher stability index values, meaning a lower stability for the specific trait and accession.

AMMI (Additive Main effect Multiplicative Interaction) analysis was performed on 24 genetic groups (GG) collected during 4 consecutive years (2020-2023). These GG comprised 11 commercial cultivars and 18 local accessions. Normalized Di stability trait values for each accession can take values between 0 and 1, being the most stable the closest to 0 (**Figure 7**). A mean Di value was also calculated for commercial and local sets revealing no significant differences between groups although individual phenotypes showed differences in every parameter performance. Among 11 commercial cultivars (‘Esperiega’, ‘Fuji’, ‘Gala Must’, ‘Gala’, ‘Golden Delicious’, ‘Pink Lady’, ‘Pinova’, ‘Reineta Gris’, ‘Reineta’, ‘Royal Gala’ and ‘Verdedoncella’), ‘Pinova’ was the most stable with a mean Di of 0.16 followed by ‘Fuji’ (0.22) and ‘Gala’ (0.23). Traditional dessert apples such as ‘Reineta’, ‘Reineta gris’ and ‘Golden delicious’ were the least stable showing Di values greater than 0.37. Although local cultivars were, in general, less stable, some exceptions were found. In particular, genotype ‘Esperiega de Ademuz’ had the lowest stability mean value (0.17), whereas the highest Di value was obtained by ‘Santa Eulalia_01’. The stability analysis has revealed that among recovered germplasm, local genotypes may challenge stability performance of well-established commercial cultivars. However, even those genotypes that do not exhibit comparable stability can still provide a great source of information for environmental studies. Furthermore, the overall mean Di values for quality traits ranged from 0.15 (fructose content) to 0.41 (TPC in peel). Notably, TPC in pulp displayed greater stability values across cultivars compared to TPC in peel. Although both traits showed instability among cultivars, ‘Pinova’ stands out as a good candidate for breeding research, as its Di values are 0.03 for TPC in pulp and 0.00 for TPC in peel. Only a few local cultivars achieved such small stability value for TPC in pulp but never simultaneously stable values for both peel and pulp. Stability values of SSC also were particularly high, indicating fluctuations in nutritional content over the years, this was especially notable in ‘Pink lady’ (Di = 0.62) for commercial cultivars, whereas ‘Reineta’ and ‘Pinova’ were the most stable genotypes. In terms of acid content, ‘Golden Delicious’ achieved one of the most stable values, although other commercial cultivars did not perform that well. That is the case of ‘Reineta gris’ and ‘Verdedoncella’ with Di of 0.67 and 0.92 respectively for malic acid (**Figure 7**). Although there were some unstable values for these parameters among local cultivars, some promising genotypes showed comparable values to more stable commercial cultivars such as ‘Manzana helada’, ‘Esperiega de Ademuz’ or ‘Ainsa_08’.

**Figure 8:**
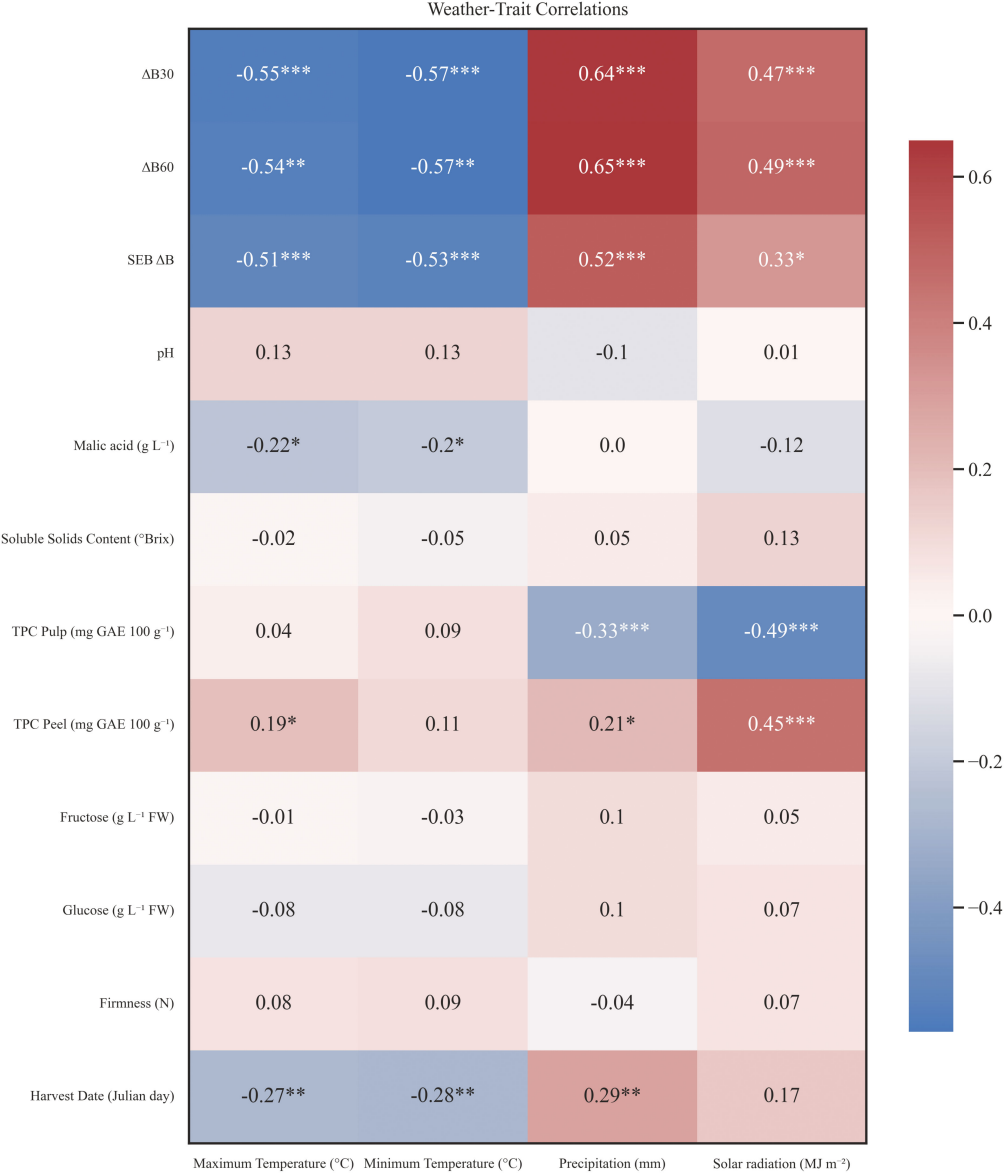
Matrix correlation between studied traits and weather conditions during 2020–2023 for 18 apple accessionsand 11 commercial cultivars. Negative correlations are shown in blue, whereas positive correlations are coloured red. Correlation significance is highlighted with a * for p < 0.05, ** for p < 0.01 and *** for p < 0.001. Studied traits consisted of 12 physicochemical parameters and weather data covered maximum and minimum temperatures, accumulated precipitation and accumulated solar radiation. Weather data was attributed to each accession from April to the specific harvest date of each case.

To understand the origin of this phenotypical variability among cultivars and years, a correlation analysis between biochemical traits and climate variables was performed (**Figure 8**). Minimum and maximum temperature, accumulated precipitation and solar radiation data were collected from April to November for each year in order to gather the apple environment conditions during fruit development and ripening. The analysis revealed significant negative correlations between Tmin (°C) and Tmax (°C) and harvest date (r = -0.27 and r = -0.28, respectively, p < 0.005), as well as with malic acid (r = -0.20 and r = -0.22 respectively, p < 0.005) and enzymatic browning indexes. Solar radiation (MJ/m^2^) correlated: positively with all enzymatic browning indexes, pointing out to an indirect connection between late varieties and enzymatic browning susceptibility; positively with TPC in peel (r = 0.45, p < 0.001); negatively with TPC in pulp (r = -0.49, p < 0.001). Likewise, accumulated precipitation (mm) showed similar correlation trends, but, additionally, correlated positively with harvest date (r = 0.29, p < 0.01). These findings highlight a potential influence of solar radiation and rainfall on fruit maturity and early browning response.

## 4 Discussion

Despite apple’s great genetic diversity, over 7500 varieties described (Szpadzik et al., 2024), commercial orchards have been reduced to a small number of productive cultivars, setting a propitious stage for cultivar genetic erosion in the future (Migicovsky et al., 2021a; Noiton and Alspach, 1996). The evolving landscape of agriculture requires holistic approaches that balance global production with shifting consumer demands, fostering innovation in nutritional trends while addressing climate change. Local genetic resources offer a broaden phenotypical landscape providing new genetical backgrounds that can differ greatly from well-established commercial cultivars and they are a source of diversity that is fundamental for having alternatives and the capacity to respond to new needs, through the search for genotypes that are better adapted to the new requirements (Castel et al., 2020; Millán-Laleona et al., 2025; Sofla et al., 2016). Our study analysed the phenotypical diversity of key traits for breeding in apple in an optimized core collection from Northeastern Spain (UPNA and CITA), previously assessed by SSR markers (Miranda et al., 2018; Pereira-Lorenzo et al., 2017).These two germplasm collections included local retrieved mountain cultivars from abandoned parcels in the northeastern area of Spain (Aragon and Navarra), alongside well-known commercial cultivars widely cultivated in Spain (Pina et al., 2014). This research provides a statistical comparison that accounts for environmental variation between collections and evaluates a trait stability tool aimed at identifying superior genotypes for breeding purposes.

Firmness and sweetness greatly determine consumer consumption preferences (Harker et al., 2008). SSC has been traditionally used as an approximate measure of sugars in apples, providing ranges in different studies of 12.10 to 16.80 °Brix in China (Tian et al., 2024), 8 to 14.64 in the Italian Campania region (Cice et al., 2023), 9.85 to 13.72 in Norway (Meland et al., 2024) or 11.2 to 14.27 in India (Kotiyal et al., 2017). Based on these results, and considering the wide SSC range obtained in our collection (10.93 to 21.20 °Brix) (**Supplementary Table S1**, **Figure 1**), we can say that the creation of the optimized germplasm core of our collection has been sufficiently representative of the diversity observable in similar collections. Moreover, titratable acidity also determines greatly cultivars’ sensory profiles and has been used together with SSC to determine SSC/TA ratio and provide a useful tool for apple subdivision into dessert, processing or cider apples (Lea, 1995). Our results showed a wide range of malic acid concentration over the collection (**Figure 1**), covering from 1.22 g/l to 17.01g/L which was smaller than the observed in an American study on fruit and juicy suitability for hard cider production (0.13 -29.67) (VanderWeide et al., 2022) but higher than the observed TA of 31 local apple cultivars in Italy (Cice et al., 2023), making the collection suitable for studies of multiple purposes.

Fruit colour is progressively developed during fruit ripening and is a limiting-factor determining consumer acceptance of fruit appearance (Chen et al., 2021; Iglesias et al., 2008). An evaluation of differences in fruit quality traits across different colour varieties was performed to detect significant statistical differences across colour groups. This analysis revealed that red apples had lower values of oxidation indexes ΔBI 30 and ΔBI 60 than yellow, green, bicoloured and brown apples. On the other hand, brown apples showed higher values of fructose (**Supplementary Figure S3**). The results may, at least partly, reflect the collection’s composition, with local cultivars—typically bicoloured or green—being more prone to browning, while commercial ones have undergone breeding for improved resistance to enzymatic browning, as they were bred for increased red skin and higher firmness (Migicovsky et al., 2021b).

Further evaluation of our collection allowed identification of trait discriminant power and characterization of trait distribution and conserved diversity (**Table 1**). Our results confirmed the adequacy of core collection selection based in observed diversity indexes with comparable or slightly lower values than similar collections (Broschewitz et al., 2024). Interestingly, a slight trend towards late-ripening cultivars was observed in the apple genotypes located at Bescós de Garcipollera (**Figure 2**), which also showed greater SSC and oxidation rate values. Consequently, these late-maturing varieties not only exhibit high nutritional value but also enhance their integration into breeding programs, which have encountered marketability challenges due to storage limitations, the need for extended availability periods and increased firmness. While other researchers have attempted to prolong the availability of local high-value cultivars by crossing early-maturing heritage varieties with commercially late-maturing counterparts to preserve nutritional quality (Sofla et al., 2016), local cultivars in our study showed a late maturing trend, which also showed increased firmness and SSC, especially at Bescos de Garcipollera, providing a genetic pool for future breeding programs with this purpose. A similar late-ripening enriched trend was observed in the REFPOP collection for Spanish orchards, when comparing similar cultivars with upper latitudes, highlighting the necessity of a more detailed analysis to determine whether the GxE effect mainly determines this fact, or a more complex interplay is playing a key role that conditions these collections distributions (Jung et al., 2020). However, our results should be interpreted with caution, as apple quality traits—such as sensory profiles (Cantin and Gracia, 2022; Sugiura et al., 2013), anthocyanin concentration (Fernández-Cancelo et al., 2022), and soil-related productivity (Baral et al., 2021; Hua et al., 2021)—are highly genotype-dependent and influenced by multiple factors. These effects can be further intensified by genotype-by-environment interactions (G × E), particularly under climate change scenarios.

Regarding bioactive substances, polyphenols, polysaccharides, organic acids and others have been found in apples and their abundance differs in pulp and peel (Patocka et al., 2020). Among these bioactive substances, polyphenols have gained special attention over the last decades, accounting for their therapeutic potential (Mansoor et al., 2023; Millán-Laleona et al., 2023, Millán-Laleona et al., 2025; Vasile et al., 2021) and availability in different plant organs and transformation during storage (Ferreira Holderbaum et al., 2010; Francini and Sebastiani, 2013; Kaeswurm et al., 2023; Vrhovsek et al., 2004). In our study, total phenolic content was assessed, revealing a great heterogeneity among studied cultivars and showing that local cultivars possessed greater content of phenolic compounds than commercial cultivars, according with previous studies (Millán-Laleona et al., 2023). Such is the case that ‘Manzana amarilla de octubre’ almost doubled the TPC concentration in pulp reported for a red-fleshed apple cultivar (Jalali et al., 2024). Although these types of red-fleshed apples have been proposed to both enhance apple TPC and improve consumers visual acceptance (Ceci et al., 2021; Kumar et al., 2022; Schmitzer et al., 2023b), our findings suggest that there is still a portion of apple’s diversity unexplored that can also account for future TPC improvement in breeding programs. Phenolic compounds’ roles in multiple processes in plant metabolism makes it a complex trait that, although it is certainly in the crosshairs of horticulture researchers due to their therapeutic potential, it has been shown in our study that TPC lacks the stability of other traits such as firmness and SSC in both commercial and local cultivars and therefore needs further studies to reduce year-to-year fluctuation, keeping in mind their potential interactions with plant metabolic processes.

Overall, observed correlations for TPC were surprising as no individual correlation can be inferred between oxidation indexes and TPC in peel nor pulp, (**Figure 3**) and contrast with the fact that TPC has been extensively reported as the main enzymatic browning determinant alongside PPO activity (Ferreira Holderbaum et al., 2010; Maioli et al., 2020; Moon et al., 2020; Tang et al., 2020). Despite the number of publications on enzymatic browning and its implications on horticultural practices (Chi et al., 2014; Chumyam et al., 2019; Fan et al., 2021; Hamdan et al., 2022; Moon et al., 2020), few studies have provided a complete phenotypical characterization of a fruit core collection. In this study, assessment of enzymatic browning was done on 111 apple genotypes, providing new data of enzymatic browning severity ranges on an optimized apple core collection, showing the great diversity of this trait on *Malus domestica*. However, our results suggest that speed rate of oxidation can be related to organic acid content (**Figure 3**). Moreover, oxidation indexes have proven to capture a great portion of genotype phenotypical diversity as they were one of the main traits with heavier loadings on the first component of the PCA (**Figure 5**). These findings corroborate that enzymatic browning is a complex polygenic trait that can involve different metabolic pathways and whose severity on apple pulp may be influenced by more factors than well-established PPO activity, results in agreement with other studies (Bielsa et al., 2025; Ferreira Holderbaum et al., 2010; Murata et al., 1995; Tang et al., 2020).

Understanding G×E is a fundamental aspect of plant breeding, as it provides insights into how different genotypes respond to diverse environmental conditions. There is evidence that the taste and textural attributes of apples have changed as a result of recent global warming (Sugiura et al., 2013). In a climate change scenario, resilient cultivars will be needed to face the challenges that may arise, therefore obtention of high-quality cultivars with stable traits under fluctuating year-to-year harvest seasons is crucial. Trait stability has been widely used in multiple species to assess yield stability and evaluate fruit quality of varieties cultivated in different environments (Cervantes et al., 2020; Dia et al., 2016; Liu et al., 2017; Ye et al., 2021). Our study revealed that among most well-known commercial cultivars in Spain, the cultivar ‘Pinova’ was the most stable, followed by ‘Fuji’ and ‘Gala’ (**Figure 7**). However, in some cases, local cultivars were more stable than commercial cultivars. Differences in stability among traits for a certain cultivar was also notable and showed a genotype-dependent pattern suggesting complex regulation of these multigenic traits. Moreover, significant correlations were found between climatic variables and studied traits. These correlations are specially concerning as they involve key marketability traits as harvest date, malic acid and TPC in pulp and peel (**Figure 8**). Strong significant negative correlations between Tmin and Tmax and harvest date were also reported in other studies (Davey et al., 2007; Mignard et al., 2021). Recently, the assessment of these effects are being studied in relation to the detection of QTLs related to genotype by environment interactions (Jung et al., 2022), establishment of reliable and efficient breeding programs (Baenziger et al., 2011) and the selection of appropriate statistical frameworks to determine the scope of the interactions (Argaw et al., 2025).

## 5 Conclusion

In this study, local recovered apple accessions have been studied alongside well-known commercial cultivars to determine the phenotypical landscape of an optimized core collection located in the Northeastern of Spain. A statistical framework was developed to evaluate the diversity comprised in the collection and the stability of measured traits over a 4-year period, providing useful information that will aid future breeding programs and provide a phenotypical background for further genetic studies. Likewise, the results highlight the intricate interplay between genetic, physiological, and environmental components, emphasizing the importance of considering higher-order interactions in predictive models and trait-based selection strategies. These findings will assist on the development of improved functional foods with better bioactive properties, the selection of stable genotypes that can accommodate to different environments, the optimization of fresh-cut products with low enzymatic browning and the selection of apple cultivars with improved sugar/acid balance and TPC content for cyder production. Future research should focus on the inherent challenges of comparing germplasm collections cultivated under contrasting environmental conditions and management practices.

## Data Availability

The original contributions presented in the study are included in the article/**Supplementary Material**. Further inquiries can be directed to the corresponding authors.

## Supplementary material

Supplementary Table 1 Complete dataset used for phenotypical analysis of 130 apple accessions and 14 commercial cultivars during 2020–2023 for CITA and UPNA germplasm collections. The dataset shows phenotypical data of assessed traits, including the difference in browning index at time 30 and 60 (ΔBI30, ΔBI60), along with oxidation speed index SEB_ΔBI, firmness, pH, malic acid content (g/L), soluble solid content (SSC), fructose (g/L) and glucose (g/L), total phenolic content (TPC) in pulp and skin, ploidy, and SSR genetic profiles for each genetic group (GG).

Supplementary Figure 1 Climate records of 2020-2023 (April to November) of the selected orchard locations from the UPNA (Pamplona) and CITA (Zaragoza and Bescos de Garcipollera) germplasm collections. Weather data include maximum temperature (°C), minimum temperature (°C), precipitation (mm) and solar radiation (MJ/m^2^).

Supplementary Figure 2 Percentage of genetic groups detected in each dataset. From left to right, all genetic groups present in the study (without selecting years), genetic groups present for both 2020 and 2021, genetic groups present during 2020-2023; genetic groups found only in one year (not enough phenotypic data to proceed with analysis).

Supplementary Figure 3 Fructose content (g/L) by peel coloration of apple genotypes. Each boxplots represent the observed fructose content for red, yellow, green, bicoloured and brown apples. Statistical differences are shown **(A, B)** between differing peel colour apples.
