# Supplementary figures and images for: Exploring phenotypic diversity and stability of key traits for apple breeding in northeastern Spanish germplasm
Source: Front Plant Sci. 2025 Sep 16;16:1623195. doi: 10.3389/fpls.2025.1623195 (PMC12479310; doi:10.3389/fpls.2025.1623195)

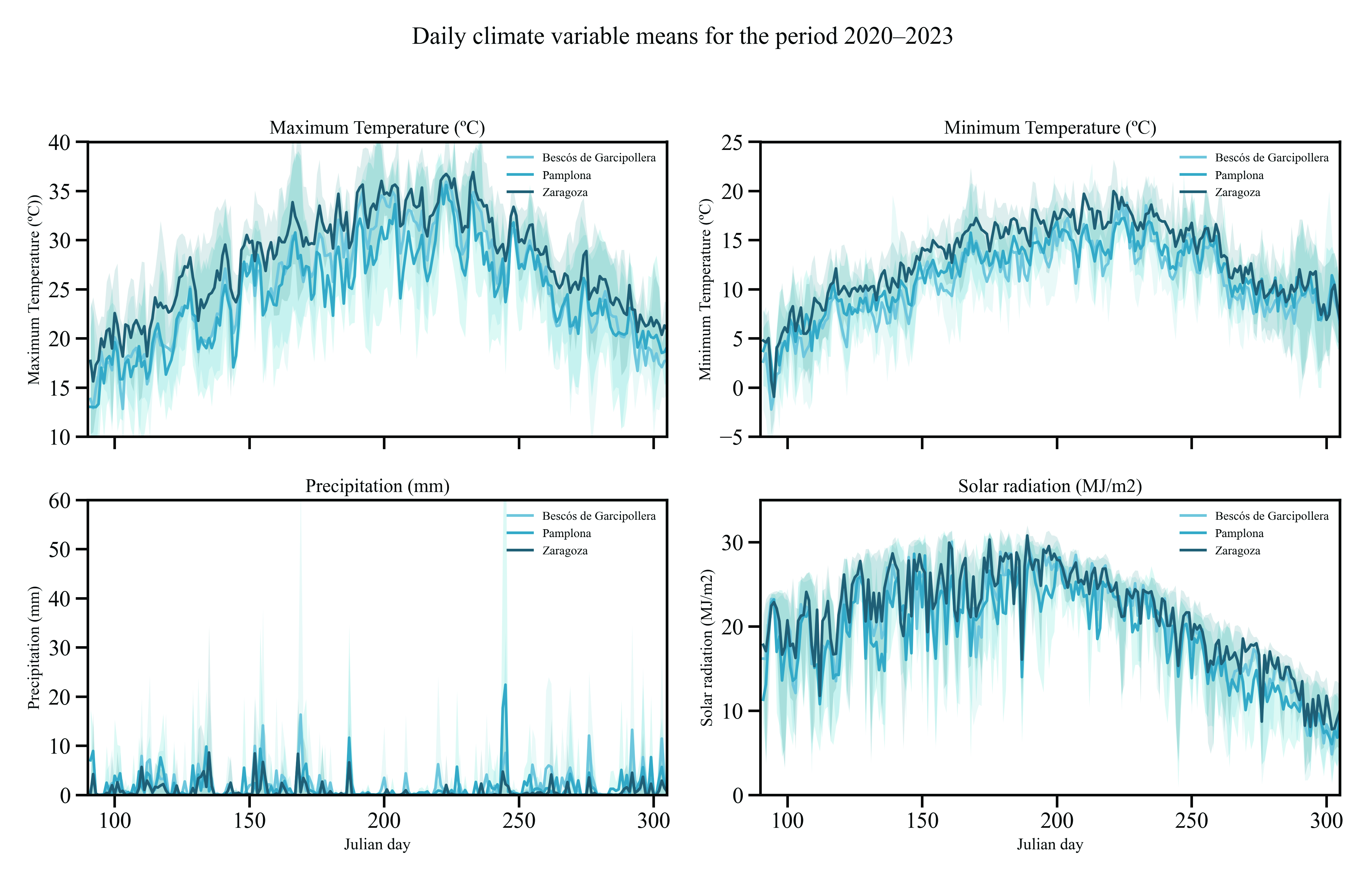

Supplement: Supplementary Figure 1 — Climate records of 2020-2023 (April to November) of the selected orchard locations from the UPNA (Pamplona) and CITA (Zaragoza and Bescos de Garcipollera) germplasm collections. Weather data include maximum temperature (°C), minimum temperature (°C), precipitation (mm) and solar radiation (MJ/m2). [file Image1.tiff]

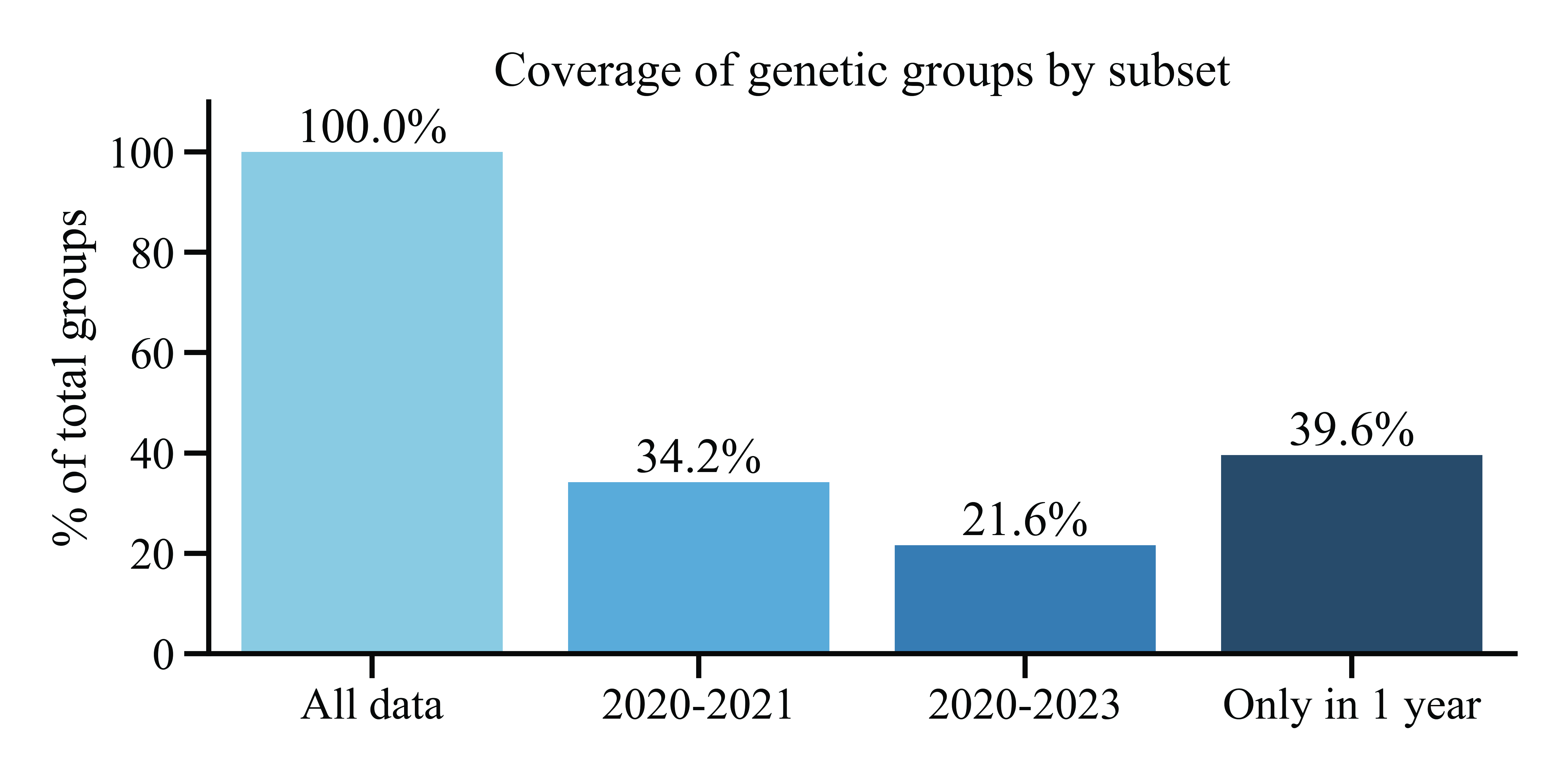

Supplement: Supplementary Figure 2 — Percentage of genetic groups detected in each dataset. From left to right, all genetic groups present in the study (without selecting years), genetic groups present for both 2020 and 2021, genetic groups present during 2020-2023; genetic groups found only in one year (not enough phenotypic data to proceed with analysis). [file Image2.tiff]

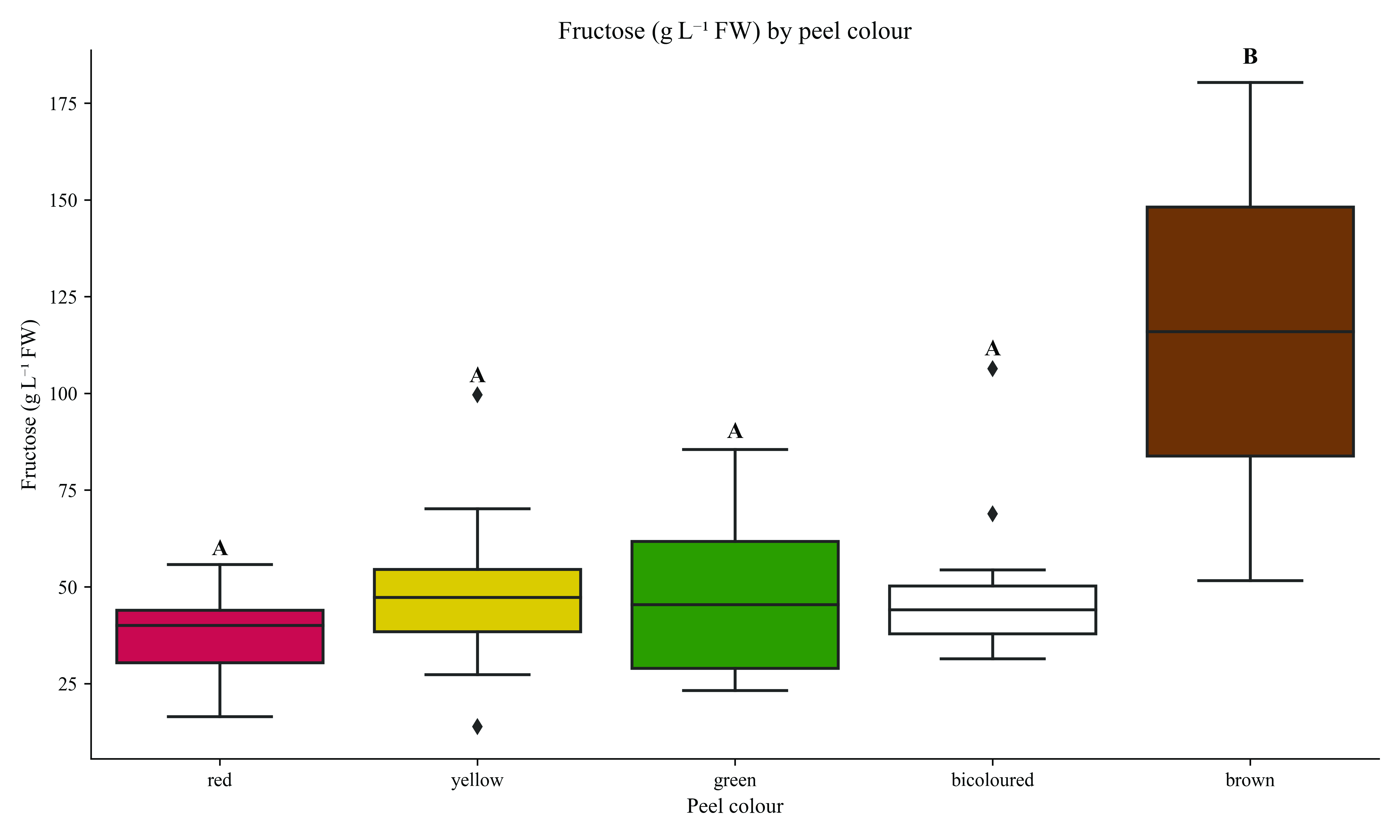

Supplement: Supplementary Figure 3 — Fructose content (g/L) by peel coloration of apple genotypes. Each boxplots represent the observed fructose content for red, yellow, green, bicoloured and brown apples. Statistical differences are shown (A, B) between differing peel colour apples. [file Image3.tiff]
